# A Loop-Mediated Isothermal Amplification (LAMP) Assay for Early Detection of *Schistosoma mansoni* in Stool Samples: A Diagnostic Approach in a Murine Model

**DOI:** 10.1371/journal.pntd.0003126

**Published:** 2014-09-04

**Authors:** Pedro Fernández-Soto, Javier Gandasegui Arahuetes, Alicia Sánchez Hernández, Julio López Abán, Belén Vicente Santiago, Antonio Muro

**Affiliations:** IBSAL-CIETUS (Instituto de Investigación Biomédica de Salamanca-Centro de Investigación de Enfermedades Tropicales de la Universidad de Salamanca), Facultad de Farmacia, Universidad de Salamanca, Salamanca, Spain; University of Melbourne, Australia

## Abstract

**Background:**

Human schistosomiasis, mainly due to *Schistosoma mansoni* species, is one of the most prevalent parasitic diseases worldwide. To overcome the drawbacks of classical parasitological and serological methods in detecting *S. mansoni* infections, especially in acute stage of the disease, development of cost-effective, simple and rapid molecular methods is still needed for the diagnosis of schistosomiasis. A promising approach is the loop-mediated isothermal amplification (LAMP) technology. Compared to PCR-based assays, LAMP has the advantages of reaction simplicity, rapidity, specificity, cost-effectiveness and higher amplification efficiency. Additionally, as results can be inspected by the naked eye, the technique has great potential for use in low-income countries.

**Methodology/Principal findings:**

A sequence corresponding to a mitochondrial *S. mansoni* minisatellite DNA region was selected as a target for designing a LAMP-based method to detect *S. mansoni* DNA in stool samples. We used a *S. mansoni* murine model to obtain well defined stool and sera samples from infected mice with *S. mansoni* cercariae. Samples were taken weekly from week 0 to 8 post-infection and the Kato-Katz and ELISA techniques were used for monitoring the infection. Primer set designed were tested using a commercial reaction mixture for LAMP assay and an *in house* mixture to compare results. Specificity of LAMP was tested using 16 DNA samples from different parasites, including several *Schistosoma* species, and no cross-reactions were found. The detection limit of our LAMP assay (SmMIT-LAMP) was 1 fg of *S. mansoni* DNA. When testing stool samples from infected mice the SmMIT-LAMP detected *S. mansoni* DNA as soon as 1 week post-infection.

**Conclusions/Significance:**

We have developed, for the first time, a cost-effective, easy to perform, specific and sensitive LAMP assay for early detection of *S. mansoni* in stool samples. The method is potentially and readily adaptable for field diagnosis and disease surveillance in schistosomiasis-endemic areas.

## Author Summary

Schistosomiasis is one of the most widespread of all human parasitic diseases, *Schistosoma mansoni* being the most important species causing human intestinal schistosomiasis. The diagnosis of the disease is mainly based on parasitological and serological methods, but they are not effective in detecting *S. mansoni* infections in the acute stage of the disease. New diagnostic tools to detect the disease during the first weeks would be desirable, permitting early treatment and preventing the pathology associated with chronic infections. An approach is the loop-mediated isothermal amplification (LAMP) technique, which can amplify DNA with high specificity and sensitivity under isothermal conditions. DNA amplification and reading of results require minimum equipment, thus the technique has great potential for use in diagnosis of neglected tropical diseases. In our study, we developed and evaluated a LAMP assay for the early detection of *S. mansoni* DNA in stool samples from mice experimentally infected with the parasite. The results indicated that our LAMP assay is specific, sensitive and cost-effective in detecting *S. mansoni* DNA in stool samples as soon as one week post-infection, when parasitological and serological methods are not effective. The assay has the potential to be developed further as a field diagnostic tool for use in schistosomiasis-endemic areas.

## Introduction

Schistosomiasis, a disease caused by parasitic worms of several species of genus *Schistosoma*, is one of the 17 neglected tropical diseases (NTDs) recognized by World Health Organization (WHO) [1]. Presently, human schistosomiasis, mainly caused by *Schistosoma mansoni* species, is one of the most widespread of all human parasitic diseases, ranking second only to malaria in terms of its socioeconomic and public health importance in developing countries in tropical and subtropical areas, especially in Sub-Saharan Africa. The disease is endemic in 74 countries infecting more than 200 million people worldwide, with 732 million people at risk of infection in known transmission areas [2], [3], [4]. On a global scale, one of thirty individuals has schistosomiasis [5]. It is also noted that the prevalence of imported schistosomiasis is increasingly a problem in non-endemic areas due to the growing number of international travelers to endemic areas, expatriates and immigrants from endemic countries [6], [7], [8].

Over time, several diagnostic techniques including parasitological and immunological methods have been tested for diagnosis of schistosome infection. As is well known, traditional parasitological methods, such as Kato-Katz assay for counting eggs in feces, are relatively inexpensive and easy to perform providing basic information on prevalence and infection intensity. However, a major limitation of these methods is their lack in sensitivity, especially in low-grade infections, as occurs in areas of low prevalence or in individuals with recent infections [9]. In addition, they are cannot be carried out in the acute phase of schistosomiasis since parasite has not started yet to lay eggs. When parasites cannot be directly detected, the immunological methods are usually applied to patients with schistosomiasis clinical signs. However, serology-based analyses currently continue to present problems, such us obtaining schistosome antigens, inability to discriminate between current or previous infection, high level of cross reactivity as well as persistence of antigens and antibodies after chemotherapy usually causing false positive results [10]. To overcome the shortcomings of both parasitological and immunological diagnostic methods, the development of new, more sensitive and specific molecular diagnostic tools for the diagnosis of schistosomiasis are desirable. In recent years, several studies have reported the application of polymerase chain reaction (PCR)-based assays for high sensible and specific detection of *Schistosoma* spp. DNA in human clinical samples, such as feces [11], [12], [13], [14], [15], sera [11], plasma [16] and urine [17]. Even though these studies have demonstrated that PCR-based technologies provided reliable, specific and sensitive tools, they are not still widely used in low-income countries because highly skilled personnel and expensive cyclers are needed. Therefore, the development of cost-effective, simple and rapid detection methods is still needed for the diagnosis of schistosomiasis.

An interesting alternative to PCR-based technologies is a molecular technique named loop-mediated isothermal amplification (LAMP). This assay is a one-step amplification reaction that amplifies a target DNA with high specificity, efficiency and rapidly under isothermal conditions [18]. LAMP employs a DNA polymerase (*Bst* polymerase) with strand-displacement activity, along with two inner primers (FIP, BIP) an outer primers (F3, B3) which recognize six separate regions within a target DNA. The auto-cycling reactions lead to accumulation of a large amount of the target DNA and other reaction by-products, such as magnesium pyrophosphate, that allow rapid visual detection and real-time measurement of turbidity [19] or visual fluorescence in the presence of fluorescent intercalating dyes [20]. Moreover, as LAMP assay is an isothermal amplification method it does not require an expensive cycler and can be performed in economical heating blocks or water baths. On this basis, simple heating methods, such as chemical heaters or thermal bottles, have been recently designed for removing the dependence upon stable electricity and allowing for LAMP to be conducted at any time in any setting [21], [22], [23], [24]. Thus, LAMP assay has all the characteristics required of realtime assays along with simple operation for easy adaptability to field conditions.

Since the LAMP assay was first reported [18], many LAMP reactions have been developed for molecular detection and diagnostics of bacterial, viral, fungal and parasitic diseases in both animals and plants [25] and their performance observed by comparing to molecular techniques, such as PCR, in order to evaluate the feasibility of LAMP technology [26], [27].

As recently reviewed by Mori et al [28], of the 17 NTDs recognized by WHO, 14 have been studied using LAMP assay, including schistosomiasis caused by *S. japonicum* in experimentally infected rabbits to evaluate the technique for early diagnosis and efficacy of chemotherapy [29], [30]. Other successfully approaches for LAMP assay to be used for *Schistosoma* spp. detection have been mainly focused in field settings for monitoring infected snails with *S. mansoni*, *S. haematobium* [31], [32] and *S. japonicum* [33], [34].

Thus, with the aim to improve in developing new, applicable and cost-effective molecular tools for the diagnosis of schistosomiasis, in our work we have developed a LAMP assay for early specific detection of *S. mansoni* in mice stool samples. In the present study, specific LAMP primer set designed was tested using different reaction mixtures each containing different *Bst* polymerases to compare results and cost-effectiveness. We also evaluated the sensitivity of the LAMP assay in comparison with classical diagnostic techniques, such as Kato-Katz and ELISA. To the best of our knowledge, this is the first report using LAMP assay for early diagnosis of active schistosomiasis in mice stool samples.

## Methods

### Ethics statement

The study protocol was approved by the institutional research commission of the University of Salamanca. Ethical approval was obtained from the Ethics Committee of the University of Salamanca (protocol approval number 48531), which approved the animal protocol. Animal procedures in this study complied with the Spanish (Real Decreto RD53/2013) and the European Union (European Directive 2010/63/EU) guidelines on animal experimentation for the protection and humane use of laboratory animals and were conducted at the accredited Animal Experimentation Facility (Servicio de Experimentación Animal) of the University of Salamanca (Register number: PAE/SA/001).

### Mice and *Schistosoma mansoni* experimental infection

Six to seven-week old female CD1 mice weighing 16–24 g (Charles River Laboratories, Barcelona, Spain) were used in the study as the source for blood and stool samples. Animals were housed at the accredited Animal Experimentation Facility of the University of Salamanca in standard polycarbonate cages and placed in humidity and temperature controlled environment with a 12 hour photoperiod and received sterilized food and water *ad libitum*. Mice were each infected with 200 *S. mansoni* cercariae which were obtained from *Biomphalaria glabrata* snails previously infected with *S. mansoni* miracidia as described elsewhere [35]. The infection was carried out following the methodology previously described by Smithers et al [36]. Uninfected mice (control group) were used as source for negative samples. All mice blood and stool samples were taken weekly from week 0 to week 8 post-infection (p.i.). Animals were monitored regularly by qualified members in animal welfare at the Animal Experimentation Facility of the University of Salamanca. Infected mice were humanely euthanised by intraperitoneal injection with pentobarbital at a 60 mg/Kg dose using 30 g needles at week 8 p.i.

### Mice samples and monitoring infection by Kato-Katz and ELISA

A total of 90 stool samples and 90 blood samples were taken from all mice throughout infection. Five stool samples as well as five blood samples were taken weekly and processed from both infected and uninfected mice groups from week 0 to week 8 p.i. Feces weekly obtained from each infected mouse was divided into two portions: one was immediately processed and examined by triplicate for counting eggs using the Kato-Katz technique [37] in a conventional microscope and another was stored at −20°C to be used afterward for DNA extraction for molecular assays. The Kato-Katz technique was used as the *gold standard* assay to pre-determine the existence of *S. mansoni* infection in stool samples. Results obtained were expressed as mean±SE. Feces weekly obtained from each non-infected mouse were kept frozen individually until DNA extraction as mentioned below.

After collection of the whole blood from each mouse in a defined time-point p.i., the sera samples were obtained by allowing the blood to clot for 15–30 minutes at room temperature and removing the clot by centrifuging at 1,000–2,000× g for 10 minutes in a refrigerated centrifuge. The resulting supernatants were immediately transferred into a clean tube and stored at −20°C until use for the evaluation of specific humoral immune response by ELISA for IgG detection. Firstly, soluble somatic extracts from adult *S. mansoni* worms (SmAg) were obtained and determined protein concentration as previously described [38]. Briefly, polystyrene microtiter plates (Costar, USA) were coated with 100 µL/well of SmAg at a protein concentration of 5 µg/mL diluted in carbonate buffer (pH 9.6). Diluted serum at 1∶100 was added to the wells and incubated for 1 h at 37°C. Horseradish peroxidase rabbit anti-mouse IgG (Sigma, USA) 1∶2000 was added. Washes were carried out three times with 200 µL of PBS-Tween 20/well. After incubation for 1 h at 37°C, substrate solution (ortho phenylene diamine+H_2_O) was added and the reaction was stopped after 10 min with 3 N H_2_SO_4_. Sera, tested by duplicate, were considered positive when the OD value exceeded the mean ± 2 SE absorbance of sera from non-infected animals.

### DNA extraction for molecular analyses

#### DNA from mice stool samples

Approximately 220–250 mg of frozen stool samples from each mouse were used for DNA extraction using the NucleoSpin Tissue Kit (Macherey-Nagel, GmbH & Co., Germany) -according to the modified protocol for DNA extraction from stool- following the manufacturers' instructions. DNA samples obtained from feces from non-infected mice were pooled and the resulting mix was treated as a single negative control sample to minimize the number of subsequent reactions.

#### Parasites DNA samples

*S. mansoni* DNA was extracted from frozen adult worms available in our laboratory using NucleoSpin Tissue (Macherey-Nagel, Germany) following the manufacturers' instructions. The concentration of *S. mansoni* DNA adult worms was measured three times by spectrophotometry using a Nanodrop ND-100 spectrophotometer (Nanodrop Technologies) to obtain an average concentration and then diluted with ultrapure water to a final concentration of 0.5 ng/µL. Subsequently, serial 10-fold dilutions from *S. mansoni* DNA thus obtained (0.5 ng/µl) were also prepared with ultrapure water ranging from 0.05 ng/µl to 0.0005 fg/µl (10^−1^ to 10^−9^) and stored at −20°C until use. DNA thus prepared was used as a positive control in all PCR and LAMP reactions as well as for assessing sensitivity of both assays.

Additionally, to determine the specificity of both PCR and LAMP assays, a total of 16 DNA samples from several helminths and protozoa were included as heterogeneous control samples. Of these samples, DNA from *Fasciola hepatica*, *Loa loa* and *Brugia pahangi* were already accessible in our laboratory. Other DNA samples were generously donated by the following: *Dicrocoelium dendriticum* and *Calicophoron daubneyi*, Y. Manga (CSIC, León, Spain); *Hymenolepis diminuta* and *Taenia taeniformis*, P. Foronda (University of La Laguna, Tenerife, Spain); *Anisakis simplex*, C. Cuéllar (UCM, Madrid, Spain) *Trichinella spiralis*, *Echinococcus granulosus*, *Cryptosporidium parvum*, *Giardia intestinalis* and *Entamoeba histolytica*, E. Rodríguez (ISCIII, Madrid, Spain) and *Schistosoma intercalatum*, A. O. Castro (CIBP-INSA, Porto, Portugal). Genomic DNA from adult male and female *Schistosoma haematobium*, Egyptian Strain, NR-31682 and genomic DNA from adult male and female *Schistosoma japonicum*, Chinese Strain, NR-36066 were obtained from the Schistosomiasis Resource Center for distribution by BEI Resources, NIAID, NIH (https://www.beiresources.org/Collection/51/Schistosome-Resource-Centers.aspx).

Concentration of these DNA samples was measured by the same method as described for *S. mansoni* DNA and then also diluted with ultrapure water to a final concentration of 0.5 ng/µL. In order to look for protein contaminations a common purity check by measuring the A_260_/A_280_ ratio was made for all samples. All these DNA samples were kept at −20°C until use in molecular assays.

### *S. mansoni* specific LAMP primers design

**Figure 1:**
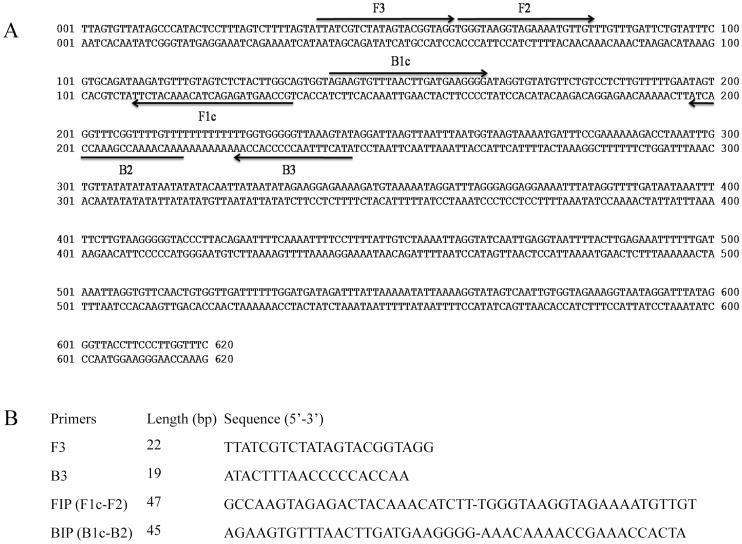
Lamp primer set targeting the selected sequence (GenBank Accesion. No. L27240) for mitochondrial *S. mansoni* minisatellite DNA region amplification. (A) The location of the LAMP primers within the selected sequence is shown. Arrows indicate the direction of extension. (B). Sequence of LAMP primers: F3, forward outer primer; B3, reverse outer primer; FIP, forward inner primer (comprising F1c and F2 sequences); BIP, reverse inner primer (comprising B1c and B2 sequences).

The first step in primers design was based on literature searches to identify potential sequences of DNA which were suspected to be used in specific detection of *S. mansoni.* Genbank sequences initially considered were tested *in silico* through BLAST searches [39] and alignment analysis. Finally, a 620 base pair (bp) sequence corresponding to a mitochondrial *S. mansoni* minisatellite DNA region was preferred and retrieved from GenBank (Accession No. L27240) [40] for the design of specific primers. Forward and backward outer primers (F3 and B3) and forward and backward inner primers (FIP: F1c-F2 and BIP: B1c-B2, respectively) were designed using the Primer Explorer V4 software (Eiken Chemical Co., Ltd., Japan; http://primerexplorer.jp/e/). Several LAMP primer sets were suggested by the software and further refinement in primer design was developed manually based on the criteria described in “A Guide to LAMP primer designing” (http://primerexplorer.jp/e/v4_manual/index.html). Specific LAMP primers sequences finally selected as well as their positions relative to the 620 bp target sequence are shown in Figure 1. All the primers were synthesized by Eurofins MWG Operon.

### PCR using outer primers F3 and B3

The outer LAMP primer pair, designated F3 and B3, was initially tested for the amplification of *S. mansoni* DNA by a *touchdown*-PCR to verify whether the correct target was amplified. The PCR assay was conducted in 25 µL reaction mixture containing 2.5 µL of 10× buffer, 1.5 µL of 25 mmol/L MgCl_2_, 2.5 µL of 2.5 mmol/L dNTPs, 0.5 µL of 100 pmol/L F3 and B3, 2 U *Taq*-polymerase and 2 µL (1 ng) of DNA template. Initial denaturation was conducted at 94°C for 1 min, followed by a touchdown program for 15 cycles with successive annealing temperature decrements of 1.0°C every 2 cycles. For these 2 cycles, the reaction was denatured at 94°C for 20 s followed by annealing at 58°C–56°C for 20 s and polymerization at 72°C for 30 s. The following 15 cycles of amplification were similar, except that the annealing temperature was 55°C. The final extension was performed at 72°C for 10 min.

The specificity of PCR using outer primers F3 and B3 was also tested with heterogeneous DNA samples from other parasites included in the study. Moreover, the sensitivity of the PCR was also assayed to establish the detection limit of *S. mansoni* DNA with 10-fold serial dilutions prepared as mentioned above. The assay was performed with 2 µL of the diluted template in each case. Negative controls (ultrapure water instead DNA template) were included. The PCR products (3–5 µL) were subjected to 2% agarose gel electrophoresis stained with ethidium bromide and visualized under UV light.

### LAMP assay

Two different reaction mixtures containing the primers designed were used to assess the LAMP assay to compare results. On one hand, the LAMP assay was performed using the *Loopamp DNA amplification Kit* (Eiken Chemicals Co., Tokyo, Japan) following manufacturers' instructions. Briefly, the reaction was carried out with a total of 25 µL reaction mixture containing 12.5 µL of 2× Reaction Mix, 40 pmol of each FIP and BIP primers, 5 pmol of each F3 and B3 primers, 1 µL of *Bst* DNA polymerase (Large fragment; LF), along with 2 µL of DNA template.

On the other hand, we tried to set up our own LAMP reaction mixture testing another *Bst* polymerase, namely *Bst* 2.0 WarmStart DNA polymerase, as well as different betaine and MgSO_4_ concentrations instead of those supplied by the commercial kit. Thus, LAMP reactions mixtures (25 µL) contained 40 pmol of each FIP and BIP primers, 5 pmol of each F3 and B3 primers, 1.4 mM of each dNTP (Bioron), 1× Isothermal Amplification Buffer −20 mM Tris-HCl (pH 8.8), 50 mM KCl, 10 mM (NH_4_)_2_SO_4_, 2 mM MgSO_4_, 0.1% Tween20- (New England Biolabs, UK), betaine (ranging 0.8, 1, 1.2, 1.4 or 1.6 M) (Sigma, USA), supplementary MgSO_4_ (ranging 2, 4, 6 or 8 mM) (New England Biolabs, UK) and 8 U of *Bst* 2.0 WarmStart DNA polymerase (New England Biolabs, UK) with 2 µL of template DNA.

To establish the standard protocol for the two LAMP reaction mixtures assayed, different temperatures were tested using a heating block (K Dry-Bath) set at 61, 63 and 65°C for 60 min and then heated at 80°C for 5 min to terminate the reaction. In each case, the optimal temperature was determined and used in the following tests. Because of the highly sensitivity of LAMP reaction, DNA contamination and carry-over of amplified products were prevented by using sterile tools at all times, performing each step of the analysis in separate work areas and minimizing manipulation of the reaction tubes. Negative controls (ultrapure water or DNA from non-infected stool samples) were included in each LAMP reaction. These controls never amplified.

#### Detection of LAMP products

After LAMP reaction, white turbidity of the reaction mixture due to the accumulation of magnesium pyrophosphate (a by-product of the reaction) was visually inspected by the naked eye. The LAMP amplification results were also visually detected by adding 2 µL of 1∶10 diluted 10,000× concentration fluorescent dye SYBR Green I (Invitrogen) to the reaction tubes. Green fluorescence was clearly observed in successful LAMP reaction, whereas it remained original orange in the negative reaction. Additionally, the LAMP reaction products (5 µL aliquots) were also monitored using 2% agarose gel electrophoresis stained with ethidium bromide, visualized under UV light and then photographed using the ultraviolet (UV) image system (Gel documentation system, UVItec, UK).

#### Specificity and sensitivity of the LAMP assay

The specificity of the LAMP assay using the two different reaction mixtures to amplify only *S. mansoni* DNA was tested against several DNA samples obtained from other parasites used as controls, including *S. haematobium*, *S. japonicum*, *S. intercalatum*, *Fasciola hepatica*, *Loa loa*, *Brugia pahangi*, *Dicrocoelium dendriticum*, *Calicophoron daubneyi*, *Hymenolepis diminuta*, *Taenia taeniformis*, *Anisakis simplex*, *Trichinella spiralis*, *Echinococcus granulosus*, *Cryptosporidium parvum*, *Giardia intestinalis* and *Entamoeba histolytica*. To determine the lower detection limit of the LAMP assay, genomic DNA from *S. mansoni* 10-fold serially diluted as mentioned above was subjected to amplification in comparison with a conventional PCR using outer primers F3 and B3.

#### Evaluation of the LAMP assay

To evaluate the ability of the LAMP assay designed to amplify *S. mansoni* DNA in real samples, we used DNA samples extracted from feces taken weekly from each experimentally infected mouse with 200 *S. mansoni* cercariae. In order to compare results, the *Loopamp DNA amplification Kit* and our *in house* LAMP reaction mixture were tested separately with all infected mice stool samples by duplicate from week 0 to week 8 p.i. In all amplification assays, a negative control (DNA mix from non-infected mice stool samples) was included.

## Results

### Monitoring of *S. mansoni* infection in mice by Kato-Katz and ELISA

The results obtained using Kato-Katz and indirect ELISA techniques on weekly stool and sera samples, respectively, from infected mice with 200 *S. mansoni* cercariae are showed in Figure S1. After infection, using the Kato-Katz technique we could only detect eggs in feces from week 6 to week 8 p.i. Specific detectable antibody levels could be measurable by ELISA from week 4 p.i. to week 8 p.i.

### Specificity and sensitivity of PCR using outer primers

**Figure 2:**
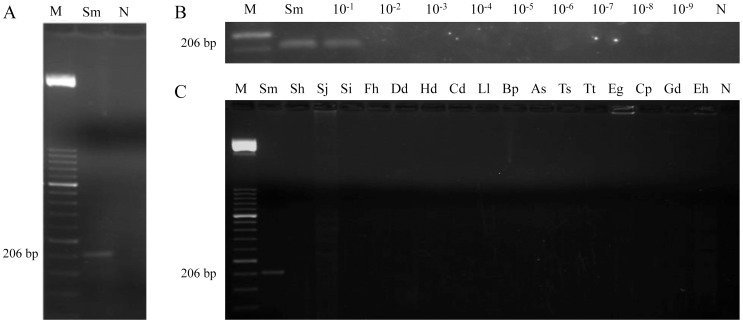
PCR verification, detection limit and specificity using outer primers F3 and B3. (A) PCR verification of expected 206 bp target length amplicon. Lane M, 50 bp DNA ladder (Molecular weight marker XIII, Roche); lane Sm, *S. mansoni* DNA (1 ng); lane N, negative control (no DNA template). (B) Detection limit of PCR. Lane M, 50 bp DNA ladder (Molecular weight marker XIII, Roche); lane Sm: *S. mansoni* DNA (1 ng); lanes 10^−1^–10^−9^: 10-fold serially dilutions of *S. mansoni* DNA; lane N, negative control (no DNA template). (C) Specificity of PCR. Lane M, 50 bp DNA ladder (Molecular weight marker XIII, Roche); lanes Sm, Sh, Sj, Si, Fh, Dd, Hd, Cd, Ll, Bp, As, Ts, Tt, Eg, Cp, Gd, Eh, *S. mansoni*, *S. haematobium*, *S. japonicum*, *S. intercalatum*, *Dicrocoelium dendriticum*, *Hymenolepis diminuta*, *Calicophoron daubneyi*, *Loa loa*, *Brugia pahangi*, *Anisakis simplex*, *Trichinella spiralis*, *Taenia taeniformis*, *Echinococcus granulosus*, *Cryptosporidium parvum*, *Giardia intestinalis* and *Entamoeba histolytica* DNA samples (1 ng/each), respectively; lane N, negative control (no DNA template).

To make sure that the expected target was amplified, a conventional PCR reaction was performed using outer primers F3 and B3 to amplify *S. mansoni* DNA. Then, a 206 bp amplicon was successful obtained (Figure 2A). In order to determine the lower detection limit of the PCR, a 10-fold serial dilution ranging from 10^−1^ to 10^−9^ of *S. mansoni* DNA was amplified. The minimum amount of DNA detectable by PCR was 0.1 ng (Figure 2B). Moreover, when DNA samples from other parasites included in the study were subjected to this PCR assay, amplicons were never obtained (Figure 2C). Additionally, when *in silico* comparisons of the expected 206 bp sequence were carried out using BLASTn searches with the currently available genomes of *S. mansoni*, *S. haematobium* and *S. intercalatum* at Wellcome Trust Sanger Institute web site (http://www.sanger.ac.uk) and *S. japonicum* at GenDB web site (http://www.genedb.org), respectively, the higher homology in alignment length, percentage of identities and E-value were obtained for *S. mansoni*. For *S. intercalatum*, *S. haematobium* and *S. japonicum*, much lower values were found (Table S1).

### Setting up LAMP assay

The optimal incubation temperature for LAMP assay using the *Loopamp DNA amplification Kit* tested with the *S. mansoni* primer set was established in a conventional heating block using a range of temperatures (61, 63 and 65°C) for 60 min to optimize the reaction conditions and then heated at 80°C for 5 min to inactivate the enzyme. The LAMP reaction could successfully take place at temperatures of 61°C, 63°C and 65°C -within the temperature range (60–65°C) recommended by the manufacturers'- although better results on agarose gels were obtained when using 63°C for amplification. Thus, the optimal temperature for LAMP using the commercial kit was established at 63°C and used for all the following applications.

To establish the standard protocol for our *in house* LAMP assay using *Bst* 2.0 WarmStart DNA polymerase we also applied a range of temperatures (61, 63 and 65°C) for testing different mixtures containing variable concentrations of betaine (ranging 0.8, 1, 1.2, 1.4 or 1.6 M) combined with supplementary variable concentrations of MgSO_4_ (ranging 2, 4, 6 or 8 mM). The best amplification results were obtained when the reaction mixture contained 1 M of betaine combined with supplementary 6 mM of MgSO_4_ (resulting a final concentration of 8 mM MgSO_4_ in 1× Isothermal Amplification Buffer) and was incubated for 60 min at 63°C in a heating block. Thereby, the reaction mixture, in addition to the specific primer set designed -hereafter SmMIT-LAMP-, was set up as the most suitable and used in all successive LAMP reactions.

**Figure 3:**
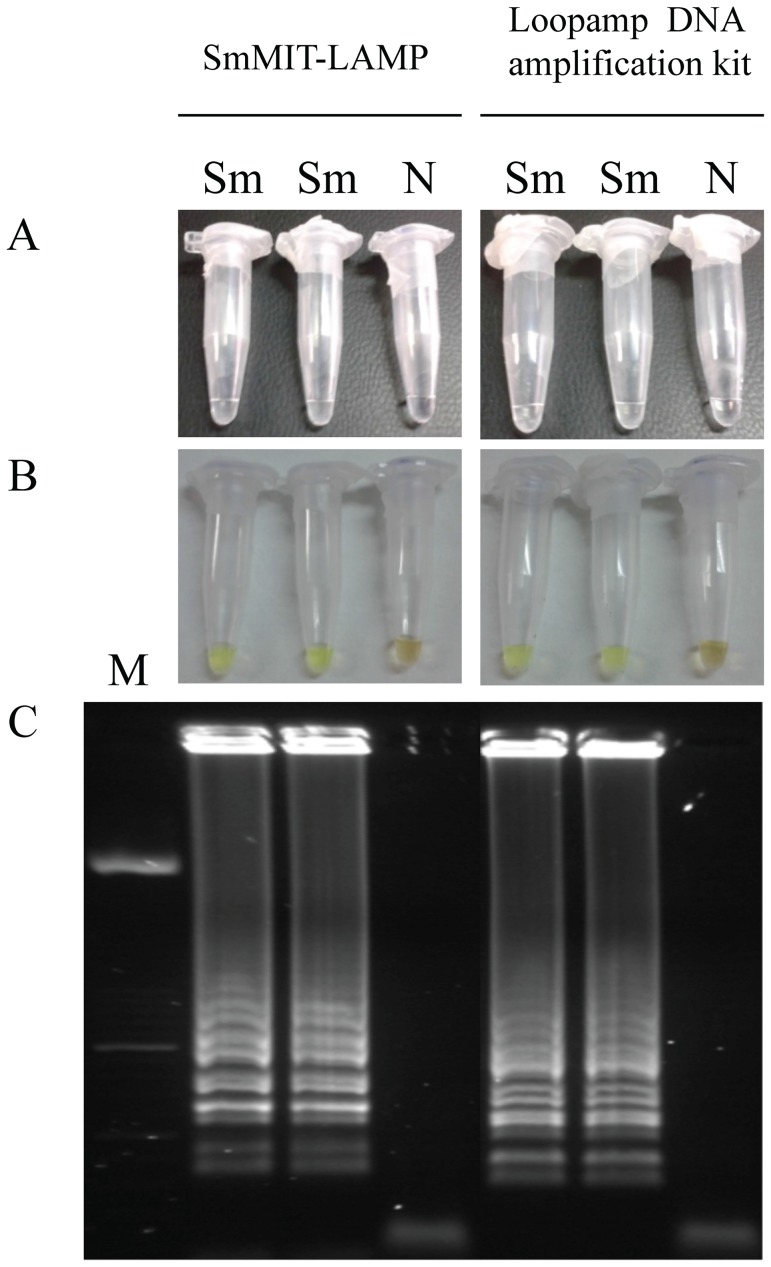
LAMP detection of *S. mansoni* genomic DNA samples using SmMIT-LAMP or the *Loopamp DNA amplification kit* at 63°C for 1 h. (A) The turbidity of the reaction mixture was inspected by the naked eye. (B) The LAMP amplification results were also visually detected by adding the fluorescent dye SYBR Green I to the reaction tubes. A successful LAMP reaction would turn to green; otherwise, it would remain orange (C) LAMP products were also monitored using 2% agarose gel electrophoresis stained with ethidium bromide. Lane M, 50 bp DNA ladder (Molecular weight marker XIII, Roche); lanes Sm: *S. mansoni* DNA (1 ng); lane N, negative control (no DNA template).

Once the most favorable conditions and molecular components were established for the two different LAMP reactions, all positive results could be visually observed by the naked eye by inspecting white turbidity as well as the color change after adding SYBR Green I. Additionally, after electrophoresis on agarose gels a ladder of multiple bands of different sizes could be also observed in positive samples (Figure 3).

### Specificity and sensitivity of LAMP assay

**Figure 4:**
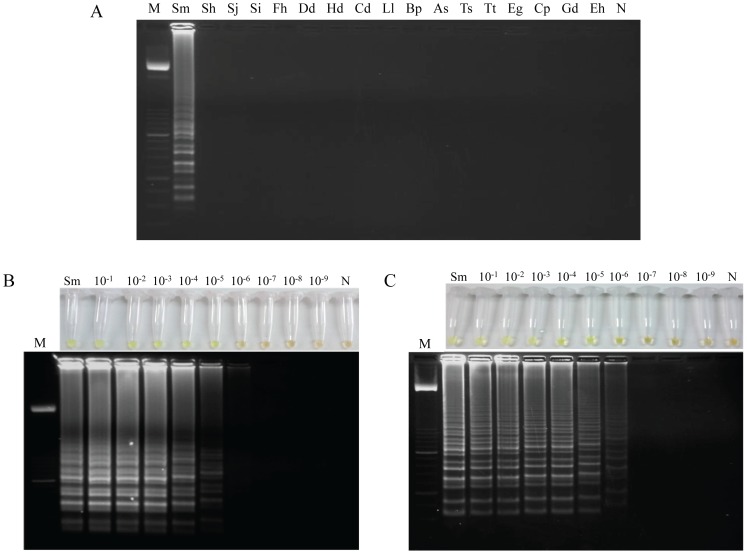
Specificity and sensitivity assessment of the LAMP assay for *S. mansoni*. (A) Specificity assessment performed with SmMIT-LAMP is shown. Identical results were obtained using *Loopamp DNA amplification Kit*. A ladder of multiple bands of different sizes could be only observed in *S. mansoni* DNA sample. Lane M, 50 bp DNA ladder (Molecular weight marker XIII, Roche); lanes Sm, Sh, Sj, Si, Fh, Dd, Hd, Cd, Ll, Bp, As, Ts, Tt, Eg, Cp, Gd, Eh, *S. mansoni*, *S. haematobium*, *S. japonicum*, *S. intercalatum*, *Dicrocoelium dendriticum*, *Hymenolepis diminuta*, *Calicophoron daubneyi*, *Loa loa*, *Brugia pahangi*, *Anisakis simplex*, *Trichinella spiralis*, *Taenia taeniformis*, *Echinococcus granulosus*, *Cryptosporidium parvum*, *Giardia intestinalis* and *Entamoeba histolytica* DNA samples (1 ng/each), respectively; lane N, negative control (no DNA template). (B) Sensitivity assessment performed with the *Loopamp DNA amplification kit* at 63°C for 1 h using serial dilutions of *S. mansoni* genomic DNA by the addition of SYBR Green I (up) or by visualization on agarose gel (down). (C) Sensitivity assessment performed with SmMIT-LAMP at 63°C for 1 h using serial dilutions of *S. mansoni* genomic DNA by the addition of SYBR Green I (up) or by visualization on agarose gel (down). Lane M: 50 bp DNA ladder (Molecular weight marker XIII, Roche); lanes Sm: genomic DNA from *S. mansoni* (1 ng); lanes 10^−1^–10^−9^: 10-fold serially dilutions; lanes N: negative controls (no DNA template).

To determine the specificity of LAMP assay for *S. mansoni*, 16 additional DNA samples from other parasites were tested for amplification. We obtained identical results using both the *Loopamp DNA amplification Kit* and SmMIT-LAMP reaction mixtures. Thus, a positive result was only obtained using DNA from *S. mansoni* whereas DNA samples from other specimens were not amplified. These results indicate that no cross-amplification was observed with these heterogeneous species in the LAMP assay, demonstrating its high specificity (Figure 4A).

Nevertheless, when sensitivity was evaluated using *S. mansoni* DNA 10-fold serially diluted, the limit of detection of LAMP using the *Loopamp DNA amplification Kit* was 10 fg (Figure 4B), whereas the limit of detection using SmMIT-LAMP was established in 1 fg (Figure 4C). These results showed that sensitivity of the SmMIT-LAMP assay is tenfold higher than that of the LAMP assay by using a standard reaction mixture supplied by the commercial kit. Furthermore, the detection limit of SmMIT-LAMP was 10^5^ times more than that previously obtained by PCR (see Figure 2B).

### Examination of mice stool samples by LAMP

**Figure 5:**
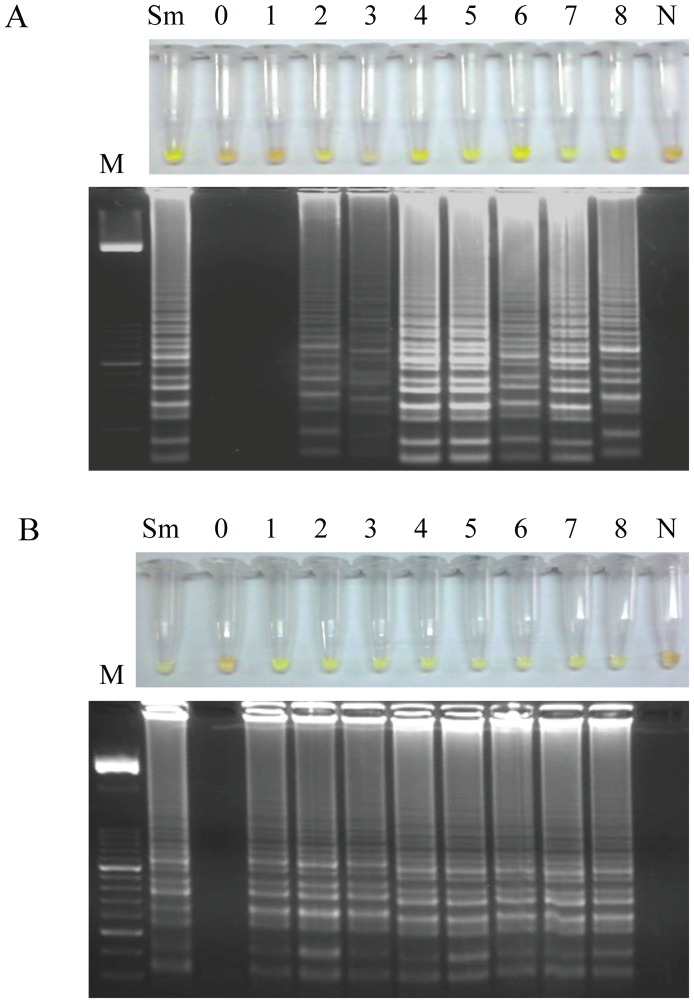
Examination of stool samples weekly obtained from mice infected with 200 *S. mansoni* cercariae. (A) By using the *Loopamp DNA amplification kit*. (B) By using the SmMIT-LAMP developed in this study. Figure shows the results obtained in feces samples weekly obtained from week 0 p.i. to week 8 p.i. from an infected mouse randomly selected. Identical results were obtained in all infected mice. Lanes M, 50 bp DNA ladder (Molecular weight marker XIII, Roche); lanes Sm, *S. mansoni* DNA, as positive control (1 ng); lanes 0–8, weeks 0, 1, 2, 3, 4, 5, 6, 7 and 8 p.i., respectively; lanes N; DNA mix from pooled DNA samples obtained from feces from non-infected mice, as negative control.

When testing stool samples from mice infected with *S. mansoni* by the *Loopamp DNA amplification Kit*, we detected positive results continuously from week 2 p.i. to week 8 p.i. in all samples analyzed (Figure 5A). By contrast, when using SmMIT-LAMP for amplification, positive results were continuously obtained in all stool samples from week 1 p.i. to week 8 p.i. (Figure 5B). Therefore, the SmMIT-LAMP assay developed was able to detect *S. mansoni* DNA in stool samples one week earlier in comparison to the LAMP assay accomplished with a standard commercial reaction mixture.

## Discussion

Human schistosomiasis, caused by several species of the trematode *Schistosoma*, is a major endemic parasitic disease in many tropical regions of Asia, Africa and South America, with *S. mansoni* being the most important species in terms of prevalence, morbidity, mortality and socioeconomic impact [2]. This morbidity and mortality is mainly associated with the chronic stage of infection, when egg deposition followed by granuloma formation in different organs, especially in liver and intestine, occurs. Although the use of praziquantel as chemotherapeutic treatment and control for the disease has a clear effect on morbidity [41], [42], resistance has been already described after repeated mass drug administration [43]. Thus, methods that allow early diagnosis, both in acute and chronic stages, are a prerequisite for effective disease control. Moreover, diagnostic tools able to detect *S. mansoni* infections mainly in acute stage would be of great value permitting early treatment that could prevent the pathology associated with chronic infections.

Currently, the *gold standard* method for diagnosis of *S. mansoni* infections is the Kato-Katz technique to count of parasite eggs excreted in feces because of its low operational costs, practicality and ability to be quantitative. However, this method requires sequential samples and is unable to detect prepatent infection, low levels of infection particularly found in children [44] or infections in individuals with a low worm burden and those in low disease transmission areas [45]. On the other hand, although many of the serology-based analyses developed present greater sensitivity than Kato-Katz technique, they currently continue to present problems in acute infections detection, lack of sensitivity, cross-reactions and false positives usually corresponding to patients who have already eliminated the parasite after efficient chemotherapy [46], [47]. All this together is currently a drawback and a considerable number of schistosomiasis patients can be incorrectly diagnosed. In this scenario, there is a need in developing new specific and more sensitive molecular diagnostic tools easy to perform in field conditions for diagnosis of schistosomiasis due to *S. mansoni*. An interesting and promising approach is the LAMP technology. Compared to PCR-based assays, LAMP has the advantages of reaction simplicity, rapidity, specificity, cost-effective and higher amplification efficiency. Furthermore, since DNA amplification and reading of results require minimum equipment, the technique has great potential for use in low-income countries [26], [27], [28]. These advantages of the technique make it appealing for use in schistosomiasis-endemic regions.

In our study, we used a *S. mansoni* murine model in order to test a new LAMP assay for early diagnosis of schistosomiasis in stool samples. Classical diagnostic techniques, such as Kato-Katz and ELISA were used for monitoring infection. Mice have been shown to be permissive to *S. mansoni* [48] and also widely used in studying dynamics of schistosome infections, including diagnosis [49]. The use of a *S. mansoni* murine model allowed us to collect well-defined stool samples that would otherwise have been difficult to obtain from human patients, such as stool samples from recently acquired infections. Thus, using the Kato-Katz technique we could detect eggs in feces collected from infected mice from week 6 to 8 p.i. When measuring specific detectable antibody levels by ELISA we could detect IgG in infected mice from week 4 p.i. until the end of the experiment. Very similar results were previously obtained by our group in detecting eggs in stool and specific antibodies in sera from mice infected with 200 *S. mansoni* cercariae [50]. As it would be logical to expect, classical diagnostic techniques were not effective to detect the acute stage of infection.

In our work, a 620 bp sequence corresponding to a mitochondrial *S. mansoni* minisatellite DNA region [40] was selected as a target for designing a LAMP-based method to detect *S. mansoni* DNA. The minisatellite region in the mitochondrial genome of *S. mansoni* seems to be unique to that species and has been already used as a target for PCR-based identification of infected snails [51]. In addition, mitochondrial sequences have some advantages over the more usual nuclear targets for amplification approaches. As each cell contains many mitochondria, multiple copies occur in every cell providing many copies of any mitochondrial DNA target region. Thus, greater sensitivity will be possible if the target sequence is present in high copy number and is highly specific and widely conserved within a particular pathogen species or group [52]. The mito-LAMP strategy has already been successfully developed for detection of several parasites, including *Opisthorchis viverrini* [53], *Trichinella spiralis* [54], *Echinococcus granulosus* [55] *and Plasmodium* spp. or *P. falciparum* specifically [56].

Once the primer set was designed, we attempted to verify the specificity for a 206 bp expected fragment amplification using a conventional PCR performed with the two outers primers to amplify *S. mansoni* DNA. As a result, a correctly sized amplicon was obtained. Additionally, no cross-reactions were found when using DNA as a target from other parasites tested in the study, including several *Schistosoma* species, such as *S. haematobium*, *S. japonicum* or *S. intercalatum*, thereby ensuring high specificity for target amplification. Furthermore, *in silico* comparisons of the expected 206 bp sequence with the *on line* available genomes of *Schistosoma* spp. showed the higher homology in alignment length with *S. mansoni*.

After verifying the specificity of the outer primers by PCR in only *S. mansoni* DNA amplification, we attempted to establish the most suitable reaction mixture for the four specific primers operation in the LAMP assay. To do this, we used a standard reaction mixture supplied by the *Loop Amplification kit* containing *Bst* DNA polymerase LF and an *in house* reaction mixture containing *Bst* 2.0 WarmStart polymerase. The latter, is an *in silico* designed homologue of *Bst* DNA polymerase LF with a reversibly-bound aptamer, which inhibits polymerase activity at temperatures below 45°C. This feature prevent about the possible undesired activity from DNA polymerases during preparation of reaction mixtures at room temperature [57], [58], [59], allowing the preparation as well as storage of the LAMP reactions for hours without changing in the final readout, as recently reported in diagnosis of brugian filariasis by LAMP using *Bst* 2.0 WarmStart polymerase [60]. It should be noted that this feature is a very important advantage in order to perform a LAMP assay in field settings where usually limited resources are found.

Regarding specificity, both LAMP reaction mixtures performed equally well at established optimal incubation temperature and exclusively *S. mansoni* DNA was amplified. However, when sensitivity of the LAMP reaction mixtures were evaluated using *S. mansoni* DNA 10-fold serially diluted, the limit of detection using SmMIT-LAMP resulted tenfold higher than that obtained using the standard reaction mixture supplied by the commercial kit (1 fg *vs.* 10 fg, respectively), thus indicating that SmMIT-LAMP is sensitive enough to detect *S. mansoni* DNA at a very low level. We underline the importance of setting up the best conditions and molecular components for primers set operation in a LAMP assay. Besides, developing an *in house* LAMP assay is much more cost-effective than using more expensive commercial kits when a large number of samples must be tested.

That increased sensitivity achieved using SmMIT-LAMP was subsequently corroborated when weekly stool samples from infected mice were tested using both LAMP reaction mixtures. In this sense, SmMIT-LAMP allowed us to detect *S. mansoni* DNA in all infected mice samples one week earlier than using the LAMP commercial reaction mixture (1 week p.i. *vs.* 2 week p.i., respectively). Therefore, an early diagnosis of active *S. mansoni* infection was possible in stool samples using SmMIT-LAMP as soon as one week p.i. It is also noteworthy that green fluorescence by adding SYBR Green I was clearly observed in all successful LAMP reactions, whereas it remained original orange in the negative reactions. This color inspection by the naked eye is a great advantage of the LAMP technique and may be preferentially used under field conditions in endemic areas without requiring electrophoresis to visualize the amplification results.

In conclusion, the results of our study demonstrated that the established SmMIT-LAMP assay is cost-effective, easy to perform, specific and sensitive enough for early detection of *S. mansoni* DNA in stool samples. Although further research for evaluation of the method for the application in patients' samples is required, the method is potentially and readily adaptable for field diagnosis and disease surveillance in schistosomiasis-endemic areas.

## Supporting Information

Figure S1 **Monitoring of** ***S. mansoni*** **experimental infection in mice by Kato-Katz and ELISA in weekly stool and sera samples, respectively, from weeks 0 to 8 post-infection.** Mice were experimentally infected with 200 *S. mansoni* cercariae. X axis represent weeks post-infection. Y axis represent number of *S. mansoni* eggs/g of feces (mean±SE; left) and absorbances of respective sera (OD) read ad 492 nm (mean±SE; right).
(TIFF)

Table S1 **BLASTn results comparing the** ***S. mansoni*** **expected 206 base pair target amplicon with the whole genome of currently available** ***Schistosoma*** **species.** *Schistosoma* species, currently genomes versions available, scaffolds, alignment situation, alignment length, identities and E-value are indicated. Results obtained for *S. mansoni, S. haematobium and S. intercalatum* come from Wellcome Trust Sanger Institute web site (http://www.sanger.ac.uk) and results obtained for *S. japonicum* come from GenDB web site (http://www.genedb.org). bp: base pair; %: percentage of identity.
(DOCX)

Checklist S1 **Checkmarks for the STARD checklist.**
(PDF)

Flow Diagram S1 **A diagram showing experimental design and results.** p.i. indicates post-infection.
(TIFF)
