# Supplementary figures and images for: A Loop-Mediated Isothermal Amplification (LAMP) Assay for Early Detection of Schistosoma mansoni in Stool Samples: A Diagnostic Approach in a Murine Model
Source: PLoS Negl Trop Dis. 2014 Sep 4;8(9):e3126. doi: 10.1371/journal.pntd.0003126 (PMC4154662; doi:10.1371/journal.pntd.0003126)

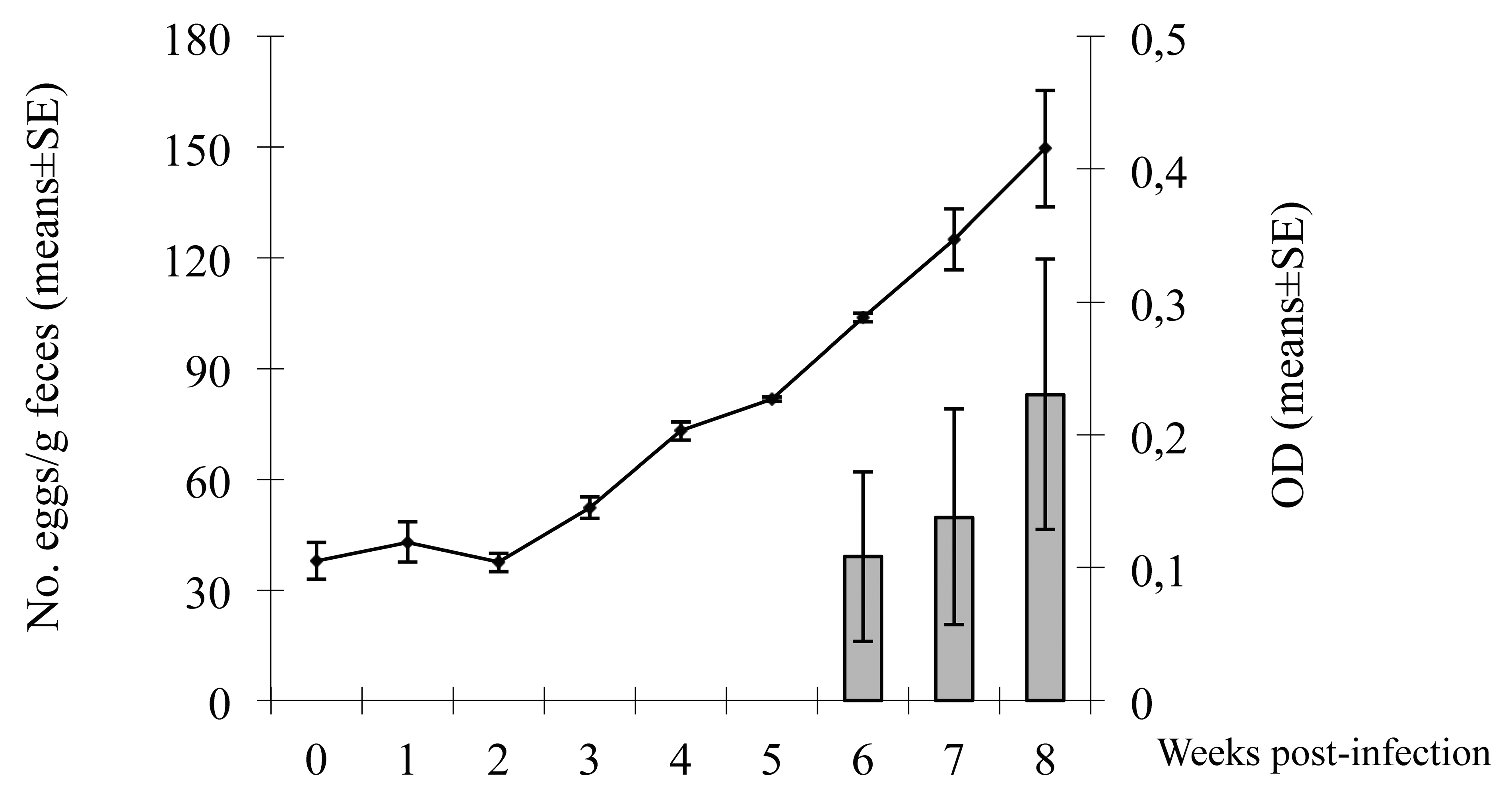

Supplement: Figure S1 — Monitoring of S. mansoni experimental infection in mice by Kato-Katz and ELISA in weekly stool and sera samples, respectively, from weeks 0 to 8 post-infection. Mice were experimentally infected with 200 S. mansoni cercariae. X axis represent weeks post-infection. Y axis represent number of S. mansoni eggs/g of feces (mean±SE; left) and absorbances of respective sera (OD) read ad 492 nm (mean±SE; right). (TIFF) [file pntd.0003126.s001.tiff]
