# Supplementary material for: A Loop-Mediated Isothermal Amplification (LAMP) Assay for Early Detection of Schistosoma mansoni in Stool Samples: A Diagnostic Approach in a Murine Model
Source: PLoS Negl Trop Dis. 2014 Sep 4;8(9):e3126. doi: 10.1371/journal.pntd.0003126 (PMC4154662; doi:10.1371/journal.pntd.0003126)
Supplement: Table S1 — BLASTn results comparing the S. mansoni expected 206 base pair target amplicon with the whole genome of currently available Schistosoma species. Schistosoma species, currently genomes versions available, scaffolds, alignment situation, alignment length, identities and E-value are indicated. Results obtained for S. mansoni, S. haematobium and S. intercalatum come from Wellcome Trust Sanger Institute web site (http://www.sanger.ac.uk) and results obtained for S. japonicum come from GenDB web site (http://www.genedb.org). bp: base pair; %: percentage of identity. (DOCX) [file pntd.0003126.s002.docx]

| *Schistosoma* spp. | Version | Scaffold | Aligment situation (bp) | Aligment lenght (bp) | Identities (%) | E value |
| --- | --- | --- | --- | --- | --- | --- |
| *S. mansoni* | *S.mansoni* genome scaffolds v.3.1 | Smp_scaff004134 | 427-630 | 206 | 93 | 4.3e^-36^ |
| *S. haematobium* | *S. haematobium* adult male (Kenya) EST sequence read | AMN2043908.q1kT3 | 122-238 | 123 | 60 | 0.11 |
| *S. japonicum* | *S. japonicum* contings GenDB version | SJC_S003670 | 21649-21821 | 176 | 60 | 0.084 |
| *S. intercalatum* | *S. intercalatum* contings v.1 | SIND_conting.78683.781 | 585-733 | 154 | 63 | 0.14 |
